# A Translocal Perspective: Mustang Images in the Cultural, Economic and Political Landscape

**DOI:** 10.3390/ani1010027

**Published:** 2010-12-14

**Authors:** Karen Dalke

**Affiliations:** Community Sciences, University of Wisconsin-Green Bay, 2420 Nicolet Drive, Green Bay, WI 54311, USA; E-Mail: dalkek@uwgb.edu; Tel.: +1-920-465-2486; Fax: +1-920-465-2791

**Keywords:** mustangs, horses, translocality, social constructions of horses

## Abstract

**Simple Summary:**

This study, based on ten years of ethnographic and archival research, explores the complexity of the mustang in the United States. Images are explored to show how one unique animal is manipulated to advance political, social and economic agendas using a theoretical framework that combines elements of praxis and globalization theory.

**Abstract:**

Translocal spaces are created out of the process of globalization whereby interventions such as electronic media and migration radically change social relations and breakdown the isomorphism of space, place, and culture [1]. This approach is useful in examining the controversy surrounding the mustang. This paper explores how different social constructions influence the management of mustangs as they move between the local and national level. At each cultural level, political, economic, and environmental issues converge encouraging the emphasis of some cultural constructions over others. These socially constructed images give insight into what the mustang means to a post-industrial culture and it may simultaneously contribute to the animal's eventual demise.

## 1. Introduction

Traditionally, culture is viewed as a physical geographical site where shared beliefs exist in a certain moment of time. Hannerz distinguishes two types of culture: territorial cultures, which are maintained by the activities of people who think and act locally in physical geographical space, and translocal cultures, which move through intersocietal space [1]. However, with increased technology and mobility, the borders have blurred. Appadurai would add that the movement or flow of people, money, technology, and ideas between cultures is increasingly important [2]. This requires a movement away from the traditional anthropological approach and movement toward the study of spaces between cultures, “non-places” like tourist zones and communities of sentiment [2,3]. The mustang and what it is imagined to be bring local cultures into the realm of translocal cultures.

The territorial culture of the American West has direct experience with mustangs, since the herd management areas exist on public lands in Arizona, California, Colorado, Idaho, Montana, New Mexico, Nevada, Oregon, Utah, and Wyoming. The mustangs and the public lands belong to the federal government and are managed by the U.S. Department of the Interior's Bureau of Land Management (BLM). However, most people will never interact with a mustang in that habitat. Most learn about mustangs through images in books, newspapers, the Internet, or postcards allowing for multiple interpretations [4].

Whatever the causes, the public's view of this spectacular animal is as diverse as the ranges they inhabit. To the wildlife purists, the wild horse is not even considered a legitimate claimant to the title of wildlife. The thoroughbred breeder and other enthusiasts of the domestic horse world see only ugly, ill-proportioned “jugheads” that have let their genes go to seed, so to speak. Those with an appreciation for history see an animal that changed the very destiny of man here and abroad. Those who are especially fond of U.S. western history see the horses as a legacy left by our forebears, both native and immigrant. The western rancher sometimes sees only a ward of the state that is eating grass that might better go to his cattle or sheep. Finally, members of Congress see the wild horse as a fact of life that generates a great deal of their mail and a species that can consume federal dollars on the same scale they can consume grass [5].

These images, removed from territorial cultures create myriad impressions that are shared by others, and a community of sentiment without specific geographic boundaries is born out of the mobility of information and people [1]. The mustang is the same species as the domestic horse, but its environment is in a constant state of flux. The BLM management of mustangs has been criticized since it began in 1971, but the issues vary across time and space [6,7] Biological or wildlife approaches to management have dominated. The importance of mustang images constructed by complex cultural, economic, and political landscapes has largely been ignored. If this diversity of images is not recognized, then it is likely that the mustang controversy will be resolved to meet primarily human economic needs. To understand the manipulation of socially constructed mustang images warrants examination of cultural, economic and political conditions.

## 2. Results and Discussion

The West as a homogenous entity is a historical remnant. In less than 100 years, there has been a great transition from primarily rural communities to large urban centers in the western part of the United States. People continue to move west for better jobs and warmer temperatures. Tourists go west to utilize the public lands in the quest for wide open spaces. There is a cultural fascination with the West as urban areas continue to swell. On some cognitive level, it seems important to know that there are wide-open spaces remaining [8].

The mustang has not changed, but the migration to a once predominantly rural West is evident (Table 1). “The West is a series of urban oases, meaning that we live in urban centers, but we have all that open space in between. The most excessive example is Nevada, which has a population of nearly two million, almost all of whom—nearly 90%—live in two urban centers, the gambling towns, Reno and Las Vegas” [9]. What the West is and what it is imagined to be conflict.

Living in an urban center or a rural community requires different normative behaviors [11,12,13,14]. In some rural communities, residents have lived by public lands prior to the Wild Free-Roaming Horse and Burro Act of 1971. To them, the mustang is part of their history. Ideas about mustang management dictated by some federal governmental office in some eastern urban center are met with suspicion. From a national perspective, public lands belong to everyone and must be managed accordingly. The mustang becomes a scapegoat in the controversy over public lands. The mustang as a historical icon is replaced by an image of a pest that stands in the way of cultural development.

Different images of the mustang are not necessarily territorially bound and can be found among long standing residents, newcomers, and tourists. Whether the mustang should be protected or eradicated has much to do with how the interested parties view land use. The focus is on perspective and not geography. Someone living in an urban setting could advocate for the mustang as they see it through the lens of protecting “natural places.” In an article about the annual Tourism Summit in Utah, Benson wrote, “People from the East Coast have no frame of reference for how big our state is and the farther away they come from, the wilder they want it…wild rivers, wild horses, wild Indians. The perception is we are not completely civilized.” [15]. For some, the perceived lack of civilization is what must be protected. In contrast, viewing the mustang as a competitor on public lands creates a less favorable image.

Images create communities of sentiment, which have translocal members. Media images and the speed by which they can be shared blur the lines between fiction and fact. As a result, viewers construct mustang images in an effort to contextualize the information creating their own realities.

**Figure 2:**
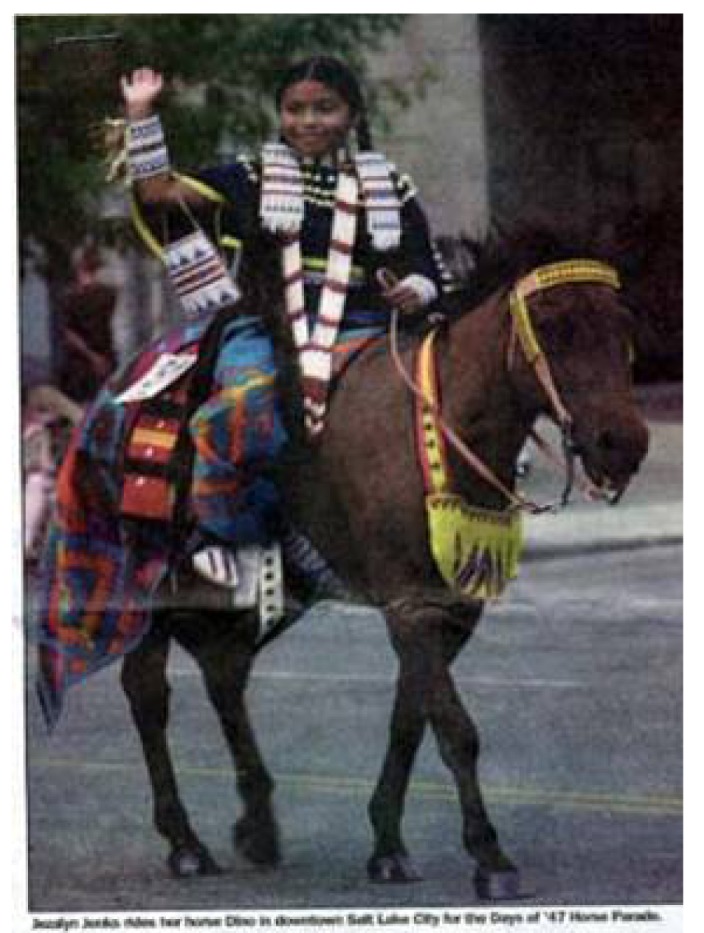
Salt Lake City's Days of '47 Parade [16].

For example, The Days of '47 Parade (Figure 2), an annual event in Salt Lake City, commemorates the pioneers' settling in Salt Lake. It also shows how local traditions become associated with states and appeal to tourists across the nation. The group of individuals who visit this event will return to their original location with images they believe to be true. Images used to convey the historical importance of mustangs are experienced with little concern for changes through time and space. This is what Appadurai is suggesting when he refers to the agency of the collective imagination [2]. The imagery created by these experiences assimilated with other knowledge about the West and mustangs maintains or alters beliefs.

People with similar interpretations form communities of sentiment with specific beliefs about mustangs and their management. Managing mustangs as if they are all alike does not acknowledge the *imagined world of the mustang* in a constantly changing cultural landscape [2]. There is a delicate balance between natural preservation and public enjoyment. Ultimately, the West changes with the people who come to see it [17,18]. The mustang cannot be managed the same way that it was in 1970. The cultural and geographic landscape has and will continue to change. Questioning the validity of images quiets discourse necessary to create solutions for the coexistence of mustangs and humans. It is important to also recognize that many mustang images are manipulated further by economic conditions.

## 3. A Changing Economic Landscape

Mustang images are manipulated to further economic agendas. In times of economic hardship, the mustang as a national cultural symbol becomes a pest. “if you can't hunt it, and you can't eat it, and you can't wear it—and I can't sell it—it should not be allowed on my public land” [6]. Emphasizing some images over others allows a rationalization of human demands. In the west, mustang images attract tourists to restaurants, hotels, and other businesses. Translocally, images are manipulated in marketing cars to advocating a lifestyle. Local communities who rely on public lands for tourism are often overlooked in translocal discourse.

**Figure 3:**
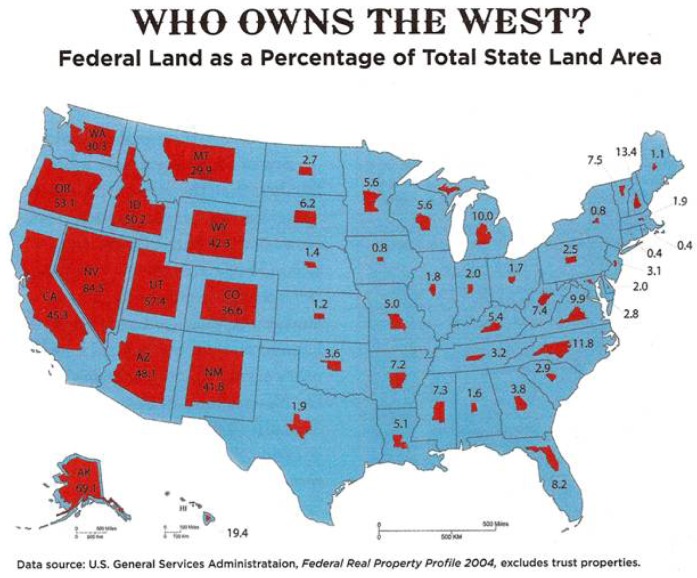
Public Land Percentages.

Tourism benefits the local economies. However, states are in a precarious position. Many western states do not own much of the land within their designated borders (Figure 3). As a result, they cannot develop public lands to benefit state residents. One of the unique features of the West is the amount of land that is public. It is federal land within state boundaries that belongs to all Americans. Since people east of the Mississippi are not confronted with this issue to the same degree, there is often a misunderstanding about public lands and mustang management further polarizing positive and negative images.

Nevada provides an excellent example regarding this issue. About 84.5% percent of land in Nevada is federally owned. Nevada is also home to more than half the mustang population. Individuals in these areas do not talk about federal land directly, although many local community members feel it is their land due to their proximity and history of caring for it—and by association, so are the mustangs. An argument over who owns the land and what should be done with it is a constant within newspaper articles. A local Nevada paper reported how two men had their cattle confiscated because they refused to pay the government grazing fees of $1.35 per month per cow and calf. Groups such as the Sagebrush Rebellion consisting of ranchers, miners, farmers, and others want less federal control [19].

The reaction to federal control seems to lie within the cultural myth of the frontier thesis that was identified by Turner more than one hundred years ago. In Turner's analysis, the East represents civilization and law; the West was more interested in personal justice devoid of finely drawn distinctions between right and wrong. Although the alleged vision of freedom is often discussed in Turner's “frontier thesis,” it is about revenge, justice, and reunion with what is natural when the East fails to provide security in times of economic and social crisis. The over-arching theme is that order in the West can be preserved without legal authority [20,21,22].

Reno and Las Vegas are no longer frontier towns, but urban centers attracting new residents from around the world. Traditional images of the mustang confront translocal needs. Federal management of public lands and mustangs, in this single most tourism-dependent state in the West, assures their survival [24]. Expansion into federal-owned properties has many barriers and those occupying polarized positions on the economic continuum will emphasize some mustang images over others.

Under the George W. Bush administration, the multiple use management of public lands tilted in favor of drilling and corporate agendas. Although local residents are often used to hasten the approval of new drilling permits, they have little say over contractors after the process has occurred [25]. Translocal communities of sentiment neutralize territorial economic agendas seeking to reduce the mustang's image as only a pest.

During the Hearings before the Subcommittee on Public Lands in 1971, Karl Weikel of Searchlight, Nevada, representing the American National Cattlemen's Association, stated:

In happier times, when there were fewer people, the ranges were open and wide. There was more room for all of us. No real problems arose. The wild horses were in essence managed by individuals, Indian tribes, and the U.S. Army Remount Service for a maximum production of usable horses. With the demise of the horse cavalry and the tremendous increase in humans as well as the advent of agriculture mechanization, the horse lost his value and became a surplus commodity in a land which demanded ever increasing production from the resources on its public lands [26].

This comment is once again relevant in the current economic climate. The mustang continues to live as it always has on federal public lands. The landscape around it has changed dramatically. The needs of the human population have used up private resources and are now looking to exploit public holdings throughout the West. The mustang, although a national heritage symbol, becomes an outdated treasure that must succumb to human economic needs.

In a 2008 documentary *Saving the American Wild Horse* proponents of alternative images suggest that the mustang acts as an indicator for the health of public lands. This argument states that mustangs are a hardy species that can survive in the most challenging environments. If mustangs are being removed from the land due to scarce water and vegetation, it is because the Bureau of Land Management is not adequately caring for the multi-use environment. Mismanaged public lands may result in not only the loss of mustangs, but have implications for human sustainability [27].

Images of the mustang are understood or manipulated in every economic landscape. The federal government manages mustangs in states where most of the land is publicly owned. Local residents use the mustang as a tourist attraction to increase utilization of local businesses. The local communities do not have economic control over the mustangs and are often subject to national economic trends. Communities of sentiment emphasize either negative or positive mustang images to further economic agendas. On the political front, these same communities can impact policy.

## 4. A Changing Political Landscape

Politics is a process whereby groups make decisions about resources. Politics occurs within territorial and intersocietal space. The idea of a static BLM or local territorial community does not exist. The mustang controversy is political and it is directly tied to imagery. Political relations contribute to the creation of mustang images. Images emerge territorially at the roundup and are transformed or maintained translocally.

## 5. Territorial Creation of Images

The roundup is more of an event than a location. Although it does physically exist for a short period of time, it is constructed to carry out a purpose (trapping horses to reduce herd size on public lands) and then is disassembled as if it never existed. During a few short days, different interest groups including local community members, animal advocates, reporters, environmentalists, wildlife enthusiasts, scientists, and BLM employees converge at this site possessing the full array of mustang images.

The roundup provides an arena for seeing how the mustang becomes politicized through images associated with history, media, and personal knowledge. The power of some groups over others determines which images, or parts of images, are retained and shared with the larger culture outside of the roundup. The roundup is also a rich context for viewing the convergence of local territorial and translocal images. There are expert participants who are familiar with the process and who pass information on to newer members to assure that the roundup can be reproduced at a later time. The roundup in this context reproduces locality in its taken-for-granted manner [2]. The local community is familiar with mustangs and knows how to round them up. History and tradition dictate decisions involving which horses will be chosen for adoption or remain on the range.

Wranglers from local areas are very familiar with the family bands within the herds on smaller ranges: smaller numbers of horses are differentiated from each other, identified by physical characteristics, and even named. As a result, the horses have a history and lineage that participants in a roundup know and share. Even BLM employees refer to the horses as “family bands” and view them as a part of the local culture. The opinions of BLM employees, on smaller ranges, depart from a strict interpretation of governmental policies. On larger ranges, the bands are bigger and are often viewed only from a distance. The horses are not as socialized to human contact because of the vastness of the range. There is no naming of the animals, and relatedness between horses is not known. The mustangs are another animal in the surrounding environment and managed as such.

Territorial images are the genesis of translocal interpretations. The law suggests that there is a standard process for wild horse removal, that decisions about wild horses are based on “hard science.” BLM employees who participate in roundups live in territorial communities where strong and opposing interest groups coexist. It is their responsibility to act fairly, manage the herd, and appear neutral. The juggling of interests they must engage in to fulfill these goals is often overlooked. The BLM is the same agency that slaughtered mustangs before they were legally protected, and it has always been viewed as a strong ally of the cattlemen, so its status among other groups concerned with the well-being of the wild horse is tenuous at best [28]. If the BLM ignores the concerns of any group, it can expect retaliation. This may explain the legal problems the agency has encountered since it undertook mustang management responsibilities. But, contravening the widespread mistrust of the BLM, bureau employees will sometimes agree with community members who are invested in particular horses. If agreeing to keep one horse on the range instead of another makes someone happy and does not result in a media blitz, the employee agrees.

The horses are categorized by different interest groups. Some simply label the mustang as an object of beauty or a cultural symbol. Others, however, distinguish agendas that represent particular political interests. Individuals tend to gravitate toward others who have similar ideas, and communities of sentiment emerge based on similar ideas, beliefs, and images of the wild horse.

Each of the communities of sentiment has justifiable arguments in favor of their political position. Those concerned with aesthetics can make the argument that wild horses are a tourist attraction that brings money to sometimes desperate economies; this group can build alliances with those who view the wild horse as a cultural symbol [29,30]. Although the legal guidelines do not address the context in which decisions are made, it is obvious that different communities of sentiment affect the BLM decision-making process.

Once trapped, the wild horse is categorized by age, conformation and color. Horses are also categorized by behavior for their intended purpose (e.g., riding, breeding, pet) or their ability to live in a domestic setting. The captured mustangs embark on a journey beyond local boundaries. Horses are moved to sanctuaries, holding areas, or adoption sites. These new material, social, and imaginative contexts maintain and transform translocal mustang images.

## 6. Translocal Images and the Politics of Placement

Although the BLM acknowledges the different images of the mustang, at the federal level it is concerned with managing them either for adoption or as well-adapted free-ranging animals. Political power allows for some images to become stronger than others. When adaptation and adoption efforts are working smoothly, the mustang is either forgotten or presented in a positive light. However, when the mustang is flooding adoption centers due to drought or is seen as a competitor on public lands, the image is negative. It is the constant interaction between territorial and translocal images that define the mustang.

The Wild Horse and Burro Act was created, in part, to resolve conflicting beliefs about the management of mustang ranges. Although the law provides protection for wild horses mandating that any person intentionally harming or killing a mustang be subject to a fine, prison sentence, or both, it cannot mandate what people believe [31]. The law that outlines the management of these wild horses does not reflect the cultural beliefs, the ever-changing environments in which they exist, or the tactical positioning and agendas by groups to present one dominant image over others. The primary focus of mustang management is animal science. Little attention is given to understanding what these horses mean to Americans outside of territorial communities.

The Act declares that the wild horses should be managed to assure a thriving ecological balance on the public lands. Individuals determining the ecological balance on these ranges may have diametrically opposed positions on land use (ranchers, wildlife managers, wild horse and burro BLM employees). To believe that everyone is working together in the best interests of the land and the animals is something that is assumed by policy makers and not supported by practice [32].

## 7. The Current Political Agenda

The Bureau of Land Management Wild Horse and Bureau Program is a diverse and geographically dispersed national agency. There is a national office in Washington D.C., a National Wild Horse and Burro Center in Sparks, Nevada, and state offices in Alaska, Arizona, California, Colorado, Idaho, Montana, Nevada, Oklahoma, Oregon, Utah, Wyoming, and Nebraska. There are also Eastern State Offices in Virginia, Mississippi, Illinois, and Wisconsin. Within each of the states where wild horses roam, there are field offices that are responsible for particular ranges in their district. The local and national offices are linked largely by electronic communication. In theory, field offices are supposed to determine the range conditions and request needed roundups to the state office, which must get national approval. However, much has changed since the 2005 Burns [33]. Stillman explains:

Two years ago, a pair of BLM scientists resigned in protest after superiors pressured them into altering the findings of a grazing study to favor the viability of cattle and other profitable grazers on the land the animals share with mustangs. Moreover, increased gas and oil drilling on public lands has put wild horses— indeed the entire range habitat—at greater risk. And a sell-off of public lands in recent years has received scant attention but dangerously reduced wildlife habitat.

The BLM, a diverse entity, manages the mustangs that belong to the American public. Trying to respond to individual interests is impossible and inefficient [35]. As a result, those communities of sentiment having the loudest voice and the most power often win the battle. Since decision making power lies in Washington D.C. and the public lands are primarily in the western United States, there is a huge disconnect for most of the American public. Most citizens learn about mustangs through a national political lens. This lens reduces the complexity of the issue and views the mustang as either good or bad.

In October 2008, BLM officials suggested that euthanasia may be the only viable alternative to deal with excess horses in holding facilities. On November 17, 2008 Madeleine Pickens, wife of billionaire T. Boone Pickens, announced she would adopt nearly 30,000 horses and burros kept in federal holding pens [36,37]. This transaction has not occurred and the discussion of what to do with these animals continues. There must be a major reform of mustang management beginning with the recognition of how we imagine the wild horse and have constructed the controversy [38].

## 8. Conclusions

The controversy over managing mustangs has little to do with the animal. An ever-changing social, economic, and political environment evokes a myriad of images. Manipulating these images furthers individual and bureaucratic agendas. How humans construct images of mustangs must be part of the discussion if long-term management of this animal is to be achieved.

In general, the current management of wild horses on public lands is assumed to be solely impacted by their legal and biological definitions although it is becoming increasingly clear that the “issues are almost entirely political, and economic, and cultural, but not scientific” [39].

This study argues that the communities of sentiment create *real* images of the wild horse shared via the Internet, television, and the movies [2]. Providing accurate and factual information does not eliminate images of the wide-open spaces of the West and the wild horses running upon them. Acknowledging the legends and cultural images is necessary for successful management of these animals.

Examining the many images of the mustang requires wading through a pool of culturally held beliefs, which are often oppositional in nature. The West has been conquered. The ability to escape and start anew in the American West, although a dominant dream, is merely a memory. The ideals of freedom and equality are challenged by urban sprawl minimizing the number of wide-open spaces that must be shared. How will Americans mediate their desire to escape to natural places while, at the same time, protecting public lands and finite resources?

The mustang is imagined in many ways, but has been managed primarily through science and technology. Although much of American culture turns to science for answers, the mustang reminds us that not all issues are logical and analytical. The wild horse demands significant attention as it highlights the importance of legends and cultural images in a society that relies heavily on science and technology to solve problems. Anthropology must play a role in emphasizing the importance of legends, imagination, and emotional responses in cultural debates about animals and the environment. It must also recognize that culture is no longer territorial, but translocal. These concepts can no longer be relegated to an inferior status. Rather, they must be regarded as part of the picture if new ways of addressing issues are to be found and the mustang is to endure for future generations.

## Figures and Tables

**Figure 1:**
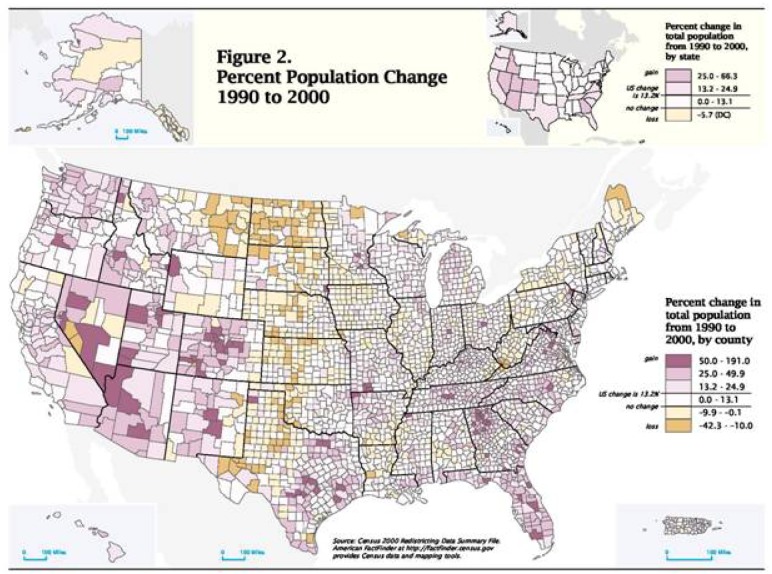
Percent change in population for U.S. States 1990–1999.

**Figure 4:**
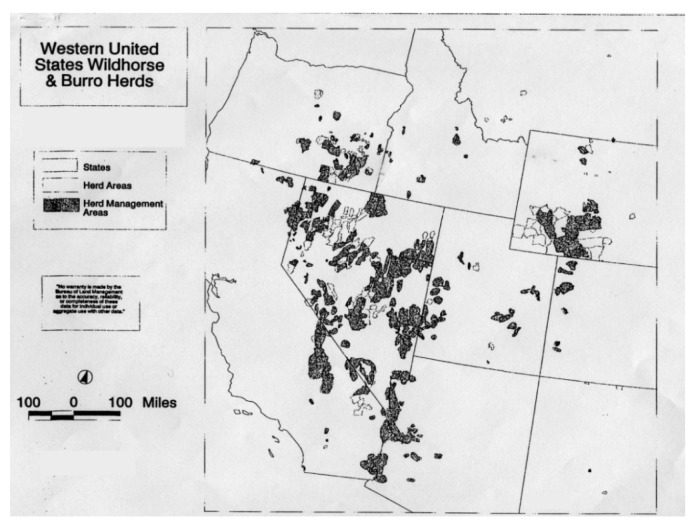
Map Highlighting Wild Horse Areas in the American West [23].

**Table 1:** Percent of urban/rural population from 1900–2000 [10].

	**1900**		**1930**		**1960**		**1990**		**2000**	
	**Urban %**	**Rural %**	**Urban %**	**Rural %**	**Urban %**	**Rural %**	**Urban %**	**Rural %**	**Urban %**	**Rural %**
**Arizona**	15.9	84.1	34.4	65.6	74.5	25.5	87.5	12.5	88.2	11.8
**California**	52.3	47.7	73.3	26.7	86.4	13.6	92.6	7.4	94.4	5.6
**Colorado**	48.3	51.7	50.2	49.8	73.7	26.7	82.4	17.6	84.5	15.5
**Idaho**	6.2	93.8	29.1	70.9	47.5	52.5	57.4	42.6	66.4	33.6
**Montana**	34.7	65.3	33.7	66.3	50.2	49.8	52.5	47.5	69.4	30.6
**N. Dakota**	7.3	92.7	16.6	83.4	35.2	64.8	53.3	46.7	55.9	44.1
**Nevada**	17	83	37.8	62.2	70.4	29.6	88.3	11.7	91.5	8.5
**Oregon**	32.2	67.8	51.3	48.7	62.2	37.8	70.5	29.5	78.7	21.3
**Utah**	38.1	61.9	52.4	47.6	74.9	25.1	87	13	88.2	11.8
**Wyoming**	28.8	71.2	31.1	68.9	56.8	43.2	65	35	65.1	34.9
